# OUTDOOR FALLS PREVENTION: PROMOTING SAFETY IN URBAN NEIGHBORHOODS

**DOI:** 10.1093/geroni/igac059.1102

**Published:** 2022-12-20

**Authors:** Tracy Chippendale

## Abstract

Outdoor falls present a significant challenge to the well-being of community dwelling older adults. There are a number of existing evidence-based programs that address fall risk, including multifactorial and exercise-based programs. However, despite the difference in risk factors for indoor and outdoor falls, no existing program specifically targets outdoor falls. To fill this gap, the Stroll Safe program was developed and refined based on a prior feasibility study. The 7-week group-based manualized program is focused on promoting safe behavioral strategies to reduce the risk of outdoor falls. In addition to presentations and community mobility coaching by the group leader, an occupational therapist, the program includes group discussion and problem solving, capitalizing on the life experiences of participants. Action planning facilitates implementation of the strategies discussed. Given that the neighborhood environment impacts both risk and fear of falling, a walkability audit (i.e. the SWAN) focused on environmental hazards is included and is used to promote awareness of hazards and as a tool for self-advocacy. Data science and machine learning facilitate the creation of resources for route planning. Vision Zero resources help to identify hotspots for pedestrian injuries, and a map of shadow is used to create a user friendly map of potential hotspots for black ice. In this symposium, we will present 1) Findings from the efficacy trial for Stroll Safe, and 2) Describe data science research that can be used to inform outdoor falls prevention programs.

